# Is preoperative IABP insertion significantly reducing postoperative complication in augmented high-risk coronary artery bypass grafting patients?

**DOI:** 10.1186/s13019-024-02925-2

**Published:** 2024-06-24

**Authors:** Faizus Sazzad, Hai Dong Luo, Guohao Chang, Duoduo Wu, Zhi Xian Ong, Theo Kofidis, Giap Swee Kang

**Affiliations:** 1 Department of Surgery, Centre for Translational Medicine, National University of Singapore, MD6, 14 Medical Drive, Singapore, 117599 Singapore; 2 Department of Cardiac, Thoracic and Vascular Surgery, National University Heart Centre, Singapore, Singapore

**Keywords:** Coronary artery bypass grafting, Intra-aortic balloon pump, Propensity score matching, In-hospital complications, Open heart surgery

## Abstract

**Background:**

The aim of this study was to determine whether pre-operative intra-aortic balloon pump (IABP) insertion improves surgical outcomes in high-risk coronary artery bypass grafting (CABG) patients.

**Methods:**

Patients with a EuroSCORE II greater than 1.2% who underwent CABG from 2009 to 2016 were included in the study, while those who utilized intra-operative or post-operative IABP were excluded. The analysis included a total of 2907 patients, with 377 patients undergoing preoperative IABP insertion (EuroSCORE II > 5.018%) and 1198 patients in the non-IABP group before matching; after propensity score matching (PSM), both groups consisted of a matched cohort of 250 patients.

**Results:**

30-day mortality events occurred in 9 (3.6%) non-IABP group and in 12 (4.8%) IABP patients (OR: 1.33 95%CI: 0.52–3.58). Kaplan-Meier survival curve analysis showed no significant differences between the two groups in mortality up to one year after the operation (*p* = 0.72). On multivariate analysis, IABP usage among the PSM patients was associated with lower 30-day mortality (OR: 0.28, 95%CI: 0.07–0.92, P-value = 0.043), 90-day mortality (OR: 0.26, 95%CI: 0.08–0.78, P-value = 0.022) and reduced risk of developing severe respiratory disorders (OR: 0.10, 95%CI:0.01–0.50, P-value = 0.011).

**Conclusion:**

Pre-operative IABP use in high-risk patients reduces 30- and 90-day mortality rates, along with a notable decrease in rates of severe respiratory disorders.

**Supplementary Information:**

The online version contains supplementary material available at 10.1186/s13019-024-02925-2.

## Introduction

Coronary artery disease surgery ranks as one of the leading causes of death globally, followed by stroke, high blood pressure, heart failure, artery diseases, and various other conditions, which may necessitate Coronary artery bypass grafting (CABG) [1]. Pre-operative Intra-aortic balloon pump (IABP) implantation was thought to reduce surgical mortality by improving hemodynamic stability and coronary perfusion [2]. However, this has become a point of contention, with newer studies observing higher mortality rates in patients receiving IABP. Notably, in the IABP-SHOCK I which is a multicentre randomized control trial, no differences in the measured hemodynamic parameters were observed between the IABP and non-IABP group [3]. This was further demonstrated in the subsequent IABP-SHOCK II trial, where no improvements in mortality and morbidity between the non-IABP and IABP group were observed up to 6 years after revascularization by either percutaneous intervention (PCI) or CABG. Moreover, comprehensive analyses involving systemic inflammation, arterial lactate, renal function, and mean arterial blood pressure have yielded inconclusive evidence, challenging the presumed pathophysiological underpinnings of IABP’s role in maintaining hemodynamic stability [4].

The discordant nature of evidence persists in studies examining the effects of pre-operative IABP insertion, with a lack of consensus regarding its impact on morbidity and mortality [5–7]. A meta-analysis encompassing 11 studies, comparing outcomes following prophylactic IABP implementation before percutaneous coronary intervention, reported no significant clinical improvements [8]. Strikingly, a retrospective study conducted by Yu et al. even suggested an increase in in-hospital mortality and morbidity associated with IABP implantation [9].

A noteworthy gap in existing research lies in the absence of studies exploring the use of pre-operative IABP within a mixed ethnic population [10–12]. Our study investigates whether prophylactic pre-operative IABP implantation among high risk patients undergoing CABG surgery contributes to reduce morbidity and mortality in the specific context of an Asian mixed-ethnicity population.

## Methods

This retrospective study was approved by the institutional review board (DSRB: 2016/01070) at the National Healthcare Group, Singapore. From 2009 to 2016, 2907 CABG patients at this tertiary institute were included. As per our institutional guidelines, preoperative IABP insertion was performed to ensure hemodynamic stability, particularly for high-risk patients identified by the EuroSCORE II. Timing might vary based on urgency, with insertion occurring the day before, on the morning of surgery a few hours before anesthesia induction, or immediately before surgery in emergencies [13].

In our series, preoperative prophylactic IABP insertion was performed in patients with severe left ventricular dysfunction, hemodynamic instability due to significant left main coronary artery disease, acute myocardial infarction, cardiogenic shock, and unstable angina. In some cases, we also utilized IABP to reduce myocardial oxygen demand and workload, mitigate postoperative complications, or serve as a bridge to decision. Maquet Datascope™ CS-100 intra-aortic balloon pumps (Getinge, Gothenburg, Sweden), with sizes of 34 cc for patients below the height of 162 cm and 50 cc for those 162 cm and above, were utilized. Post-insertion, X-rays verified proper positioning just distal to the aortic arch. Comprehensive patient data was documented and stored in the department registry.

### Analysis cohort

**Fig. 1:**
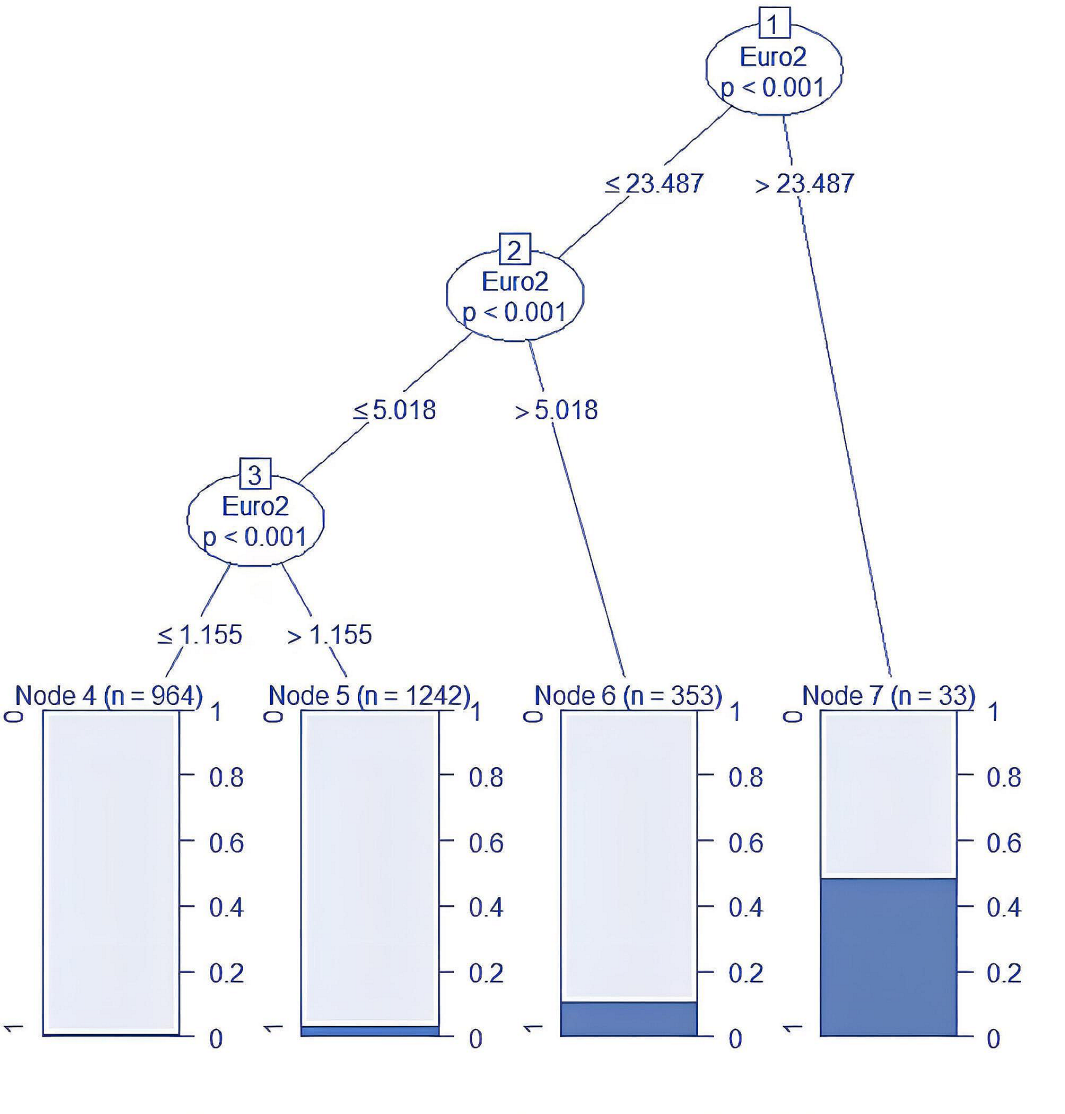
Chi-square automatic interaction detection (CHAID) Decision Tree analysis showing the steps of determination of the “high-risk” group; it was identified based on a EuroSCORE II greater than 5.018%

High mortality risk was defined by a EuroSCORE II > 5.018%, determined through a Chi-square automatic interaction detection (CHAID) Decision Tree analysis in R Studio, as depicted in Fig. 1. Patients were then stratified into high and low-risk groups based on EuroSCORE II and one-year all-cause mortality rates. This approach was employed due to considerations that the EuroSCORE II cut-offs recommended by the American Heart Association for predicting mortality [14, 15] were suboptimal for a mixed Asian population [16, 17]. IABP was inserted for patients classified as high-mortality risk based on the EuroSCORE II and the institutional IABP insertion protocol, labeling these patients as the “intervention” or “IABP group.” Patients with a EuroSCORE II of < 5.018% who did not receive an IABP were labeled as the “non-IABP” group [18]. Exclusion criteria encompassed patients receiving intra-operative or post-operative IABP support, those at low mortality risk with a EuroSCORE II of < 1.2%, and individuals with incomplete information.

### Definitions of study outcomes

This study was aimed primarily at evaluating all-cause mortality over a one-year follow-up period. The primary outcomes include short-term mortality assessed using 30-day mortality events and long-term mortality analyzed through Kaplan-Meier survival curve analysis. Secondary outcomes encompassed in-hospital events such as post-operative renal replacement therapy (RRT), cerebrovascular accidents, severe respiratory disorders, disabling complications (necessitating additional medical interventions, rehabilitation, and support to manage their consequences), septicemia, infection, and the postoperative length of stay.

### Propensity score matching analysis

We employed propensity score matching (PSM) to simulate a randomized trial. We estimated the propensity score for each individual by using logistic regression to model the probability of receiving IABP given the variables shown in Table 1. Subsequently, IABP patients were matched to the non-IABP group at a 1:1 ratio using the “nearest-neighbor” method, with a matching caliper set at 0.25 × standard deviation. Individuals with propensity scores that did not permit matches within the caliper were excluded. A total of 250 pairs of the IABP and the non-IABP group patients were successfully matched, and the adequacy of the match was assessed by ensuring that all baseline covariates exhibited a standardized mean difference of < 10% (Table 1). The matching procedure was executed using R Studio (Integrated Development for R, Boston, MA) [19].

**Table 1:** Comprehensive overview of the patient population under consideration

	Before PSM			After PSM		
	Non-IABP	IABP	*p*-value	Non-IABP	IABP	*p*-value
N	1198	377		250	250	
Age (median [IQR])	65.0 [58.0, 71.0]	61.0 [55.0, 69.0]	< 0.001	62.0 [55.0, 68.8]	63.0 [55.3, 69.0]	0.585
Male, n (%)	904 (75.5)	304 (80.6)	0.045	200 (80.0)	192 (76.8)	0.447
Race, n (%) [Chinese]	795 (66.4)	245 (65.0)	0.151	171 (68.4)	168 (67.2)	0.950
BMI (median [IQR])	24.0 [21.8, 26.8]	24.5 [22.3, 27.1]	0.035	24.2 [22.3, 26.9]	24.4 [21.9, 27.3]	0.897
Pre-operative RRT, n (%)	81 (6.8)	311 (2.9%)	0.008	7 (2.8%)	8 (3.2%)	1.000
Family history of CAD, n (%)	65 (5.4)	23 (6.1)	0.846	19 (7.6)	17 (6.8)	0.793
Pulmonary hypertension, n (%)	26 (2.2)	10 (2.7)	0.727	10 (4.0)	8 (3.2)	0.81
Diabetes management, n (%) [Oral therapy]	460 (38.4)	121 (32.1)	< 0.001	92 (36.8)	87 (34.8)	0.809
Morbid obesity, n (%)	16 (1.3)	2 (0.5)	0.315	2 (0.8)	2 (0.8)	1.000
Last creatinine level (median [IQR])	92.0 [75.0, 121.0]	85.0 [70.0, 114.0]	0.002	88.5 [74.3, 112.0]	84.0 [71.0, 112.5]	0.224
Hypertension, n (%) [Treated or BP greater than 140/90]	1030 (86.0)	273 (72.4)	< 0.001	59 (23.6)	51 (20.4)	0.654
Peripheral vascular disease, n (%)	173 (14.4)	25 (6.6)	< 0.001	21 (8.4)	19 (7.6)	0.869
Carotid disease, n (%)	146 (12.2)	33 (8.8)	0.082	24 (9.6)	29 (11.6)	0.561
Extracardiac arteriopathy, n (%)	284 (23.7)	55 (14.6)	< 0.001	40 (16.0)	45 (18.0)	0.634
Extensive atherosclerosis, n (%)	129 (10.8)	35 (9.3)	0.468	16 (6.4)	22 (8.8)	0.399
Previous percutaneous intervention, n(%)	247 (20.6)	76 (20.2)	0.905	48 (19.2)	40 (16.0)	0.411
Inotropic support, n (%)	5 (0.4)	42 (11.1)	< 0.001	4 (1.6)	5 (2.0)	1.000
Ventilated preoperatively, n (%)	7 (0.6)	36 (9.5)	< 0.001	5 (2.0)	6 (2.4)	1.000
Cardiogenic shock, n (%)	18 (1.5)	56 (14.9)	< 0.001	12 (4.8)	15 (6.0)	0.692
Hemodynamic instability, n (%)	10 (0.8)	53 (14.1)	< 0.001	6 (2.4)	11 (4.4)	0.324
Cardiomegaly, n (%)	8 (0.7)	3 (0.8)	0.186	1 (0.4)	1 (0.4)	0.919
Number of previous MI, n (%) [None]	410 (34.2)	78 (20.7)	< 0.001	62 (24.8)	59 (23.6)	0.964
Congestive heart failure, n (%)	276 (23.0)	132 (35.0)	< 0.001	66 (26.4)	73 (29.2)	0.549
Angina, n (%) [Unstable]	159 (13.3)	190 (50.4)	< 0.001	83 (33.2)	94 (37.6)	0.555
Resus, n (%) [Yes]	16 (1.3)	35 (9.3)	< 0.001	10 (4.0)	11 (4.4)	1.000
Preoperative heart rhythm, n (%) [AF]	53 (4.4)	19 (5.0)	< 0.001	10 (4.0)	13 (5.2)	0.868
Dyspnoea, n (%) [≥ 3]	130 (10.9)	91 (24.2)	< 0.001	40 (16)	43 (17.2)	0.933
Extent of coronary artery disease, n (%) [Three vessels with > 50% stenosis]	957 (79.9)	296 (78.5)	0.042	200 (80.0)	206 (82.4)	0.143
LMS CAD, n (%) [≥ 51% stenosis]	305 (25.5)	230 (61)	< 0.001	133 (53.2)	131 (52.4)	0.929
Ejection fraction (median [IQR])	50.0 [38.0, 60.0]	40.0 [30.0, 55.0]	< 0.001	45.0 [35.0, 55.0]	44.5 [30.5, 55.0]	0.535
Diastolic dysfunction, n (%)	435 (36.3)	132 (35.0)	0.692	85 (34.0)	83 (33.2)	0.925
RWMA, n (%) [Yes]	785 (65.5)	264 (70.0)	0.12	174 (69.6)	172 (68.8)	0.923
Redo-operation, n (%)	7 (0.6)	3 (0.8)	0.937	3 (1.2)	2 (0.8)	1.000
Operative urgency, n (%)			< 0.001			0.914
Elective	694 (57.9)	58 (15.4)		54 (21.6)	51 (20.4)	
Emergency	67 (5.6)	87 (23.1)		36 (14.4)	36 (14.4)	
Salvage	0 (0.0)	7 (1.9)		0 (0.0)	0 (0.0)	
Urgent	437 (36.5)	225 (59.7)		160 (64.0)	163 (65.2)	
CABG Category, n (%)			0.001			0.901
Off-pump isolated CABG	74 (6.2)	5 (1.3)		3 (1.2)	3 (1.2)	
On-pump beating heart isolated CABG	54 (4.5)	18 (4.8)		9 (3.6)	11 (4.4)	
On-pump isolated CABG	1070 (89.3)	354 (93.9)		238 (95.2)	236 (94.4)	

AF = Atrial fibrillation/flutter; BMI = Body mass index; CAD = coronary artery disease; CABG = Coronary artery bypass graft; IQR = Inter quartile range; LMS = Left main stem; MI = Myocardial infarction; RRT = Renal replacement therapy; RWMA = Regional wall motion abnormality; VT or VF = Ventricular tachycardia or Ventricular fibrillation;

### Statistical analysis

In the pre-matched cohort, categorical variables were expressed as frequencies and percentages. Normality of continuous variables was assessed using Shapiro-Wilk’s method. Normally distributed continuous variables were presented as mean and standard deviations, while non-normally distributed ones were represented as median and inter-quantile range. Stratification by IABP usage allowed comparisons using the Mann-Whitney U test for continuous variables and the Chi-square test for categorical variables. Paired univariate analysis was conducted for the two groups. Categorical variables were compared using McNemar’s Test, and continuous variables were compared using paired T test. Kaplan-Meier survival curve analysis was performed using the signed-rank test. All statistical analyses were carried out using R Studio, with a significance threshold set at p-value < 0.05 [19].

## Results

This retrospective study focused on patients undergoing CABG between 2009 and 2016. The patient cohort, consisting of 2907 individuals, was refined by excluding those who received intra-operative or post-operative IABP support, low-risk patients (EuroSCORE II < 1.2%), and those with missing information. The final pre-matched cohort comprised 1575 individuals, categorized into 1198 the non-IABP group and 377 pre-operative IABP patients group (Supplementary Fig. 1). After PSM matching, both groups consist of a matched cohort of 250 patients.

Table 1 presents a comprehensive overview of patient characteristics both before and after PSM. Prior to PSM, patients with pre-operative IABP insertion exhibited higher median age, male predominance, body mass index (BMI), serum creatinine levels, and ejection fractions. All patient characteristics were generally elevated in the pre-operative IABP group, with some exceptions such as preoperative RRT, diabetes, hypertension, peripheral vascular disease, and extracardiac arteriopathy.

After PSM, the matching protocol successfully balanced baseline covariates between the two groups, as indicated by standardized mean differences < 10%. No significant differences were found in patient characteristics post-matching, ensuring a more comparable and balanced comparison between the non-IABP and pre-operative IABP patients group (Supplementary Fig. 2). These findings provided a robust foundation for subsequent analyses and interpretations of the study outcomes.

### Study outcomes

#### Mortality

**Fig. 2:**
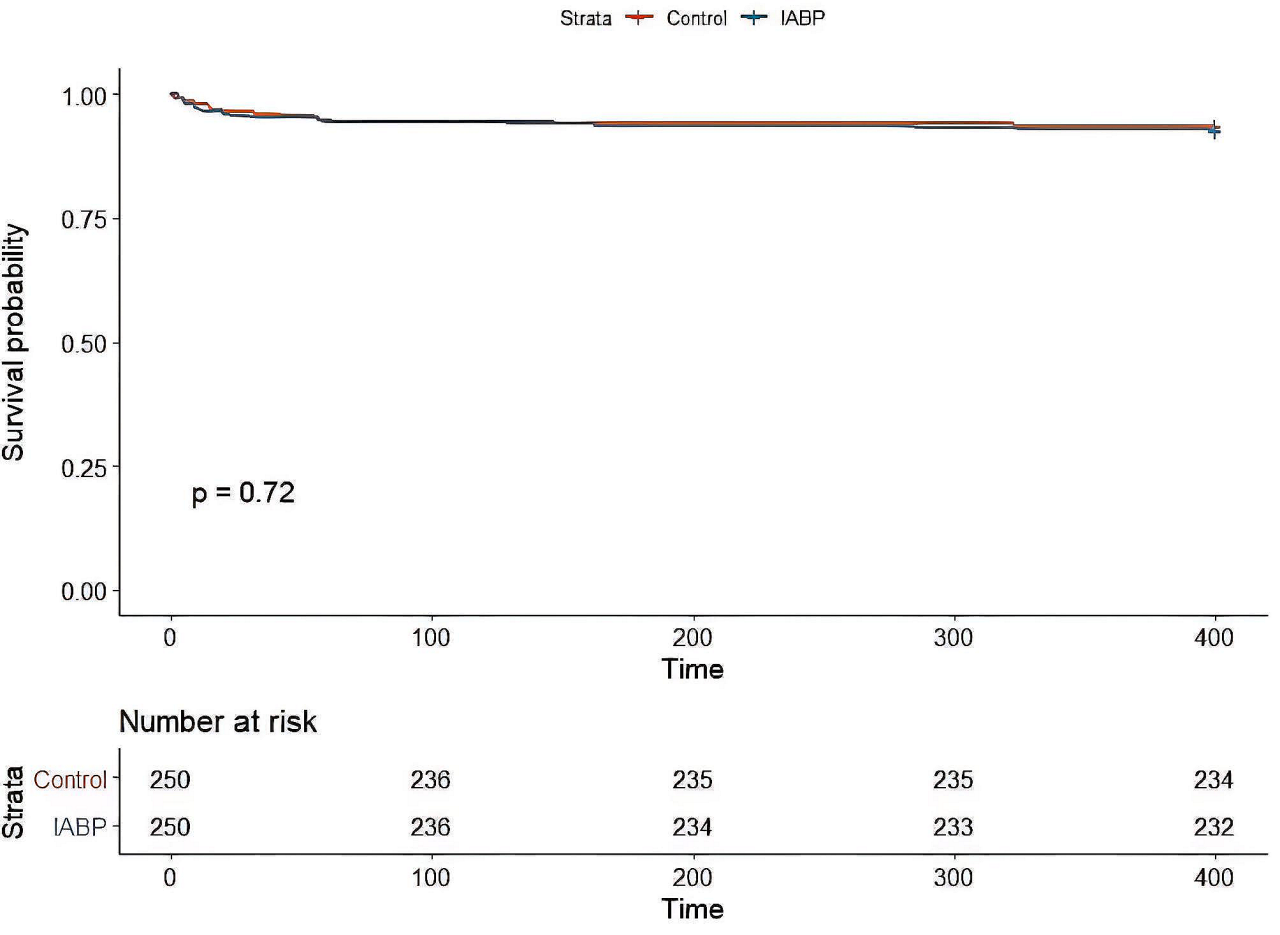
The Kaplan-Meier survival curve shows the probability of survival over time for patients who underwent propensity-score-matched IABP usage compared to the non-IABP group after CABG. The curve indicates that there were no significant differences in mortality rates between the two groups up to one year after the operation, as evidenced by the overlapping survival curves

**Fig. 3:**
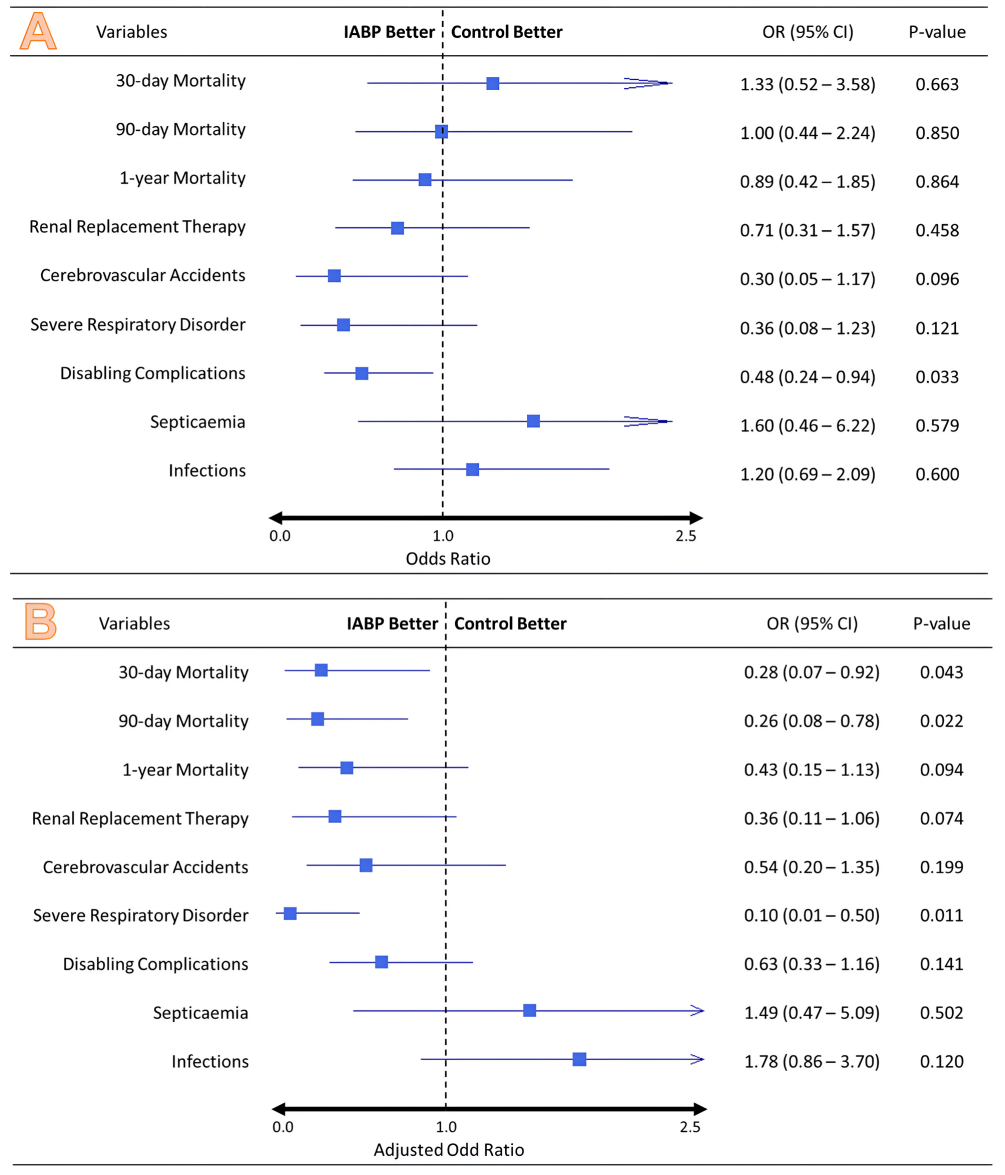
Forest plot showing (**A**) Mcnemar’s test – IABP usage among the PSM patients had better outcomes for 1-year mortality, Renal replacement therapy, Cerebrovascular accidents, Severe respiratory disorders, and disabling complications (**B**) multivariate analysis result on IABP usage among the PSM patients was associated with lower 30-day mortality and 90-day mortality rates, in addition to better 1-year mortality, Renal replacement therapy, Cerebrovascular accidents, Severe respiratory disorders, and disabling complications. (*If the upper limit of 95% CI exceeds 2.5, it is represented by an arrow.)

Table 2 presents the mortality rates in the PSM cohort, revealing no significant differences in 30-day, 90-day, and 1-year mortality rates between the groups. Specifically, 30-day mortality occurred in 9 (3.6%) the non-IABP group and 12 (4.8%) IABP patients group, with an odds ratio (OR) of 1.33 (95%CI: 0.52–3.58). Kaplan-Meier survival curve analysis (Fig. 2) indicated no noteworthy disparities in mortality up to one-year post-operation (*p* = 0.72). In the multivariate analysis, IABP usage in the PSM group demonstrated an association with lower 30-day mortality (OR: 0.28, 95%CI: 0.07–0.92, P-value = 0.043) and 90-day mortality (OR: 0.26, 95%CI: 0.08–0.78, P-value = 0.022) rates (Fig. 3).

**Table 2:** Primary and secondary outcomes of the study

**Primary Outcomes**	**Non-IABP**	**IABP**	**OR (95% CI)**	***p***-**value**
30-day mortality	9 (3.6%)	12 (4.8%)	1.33 (0.52–3.58)	0.663
90-day mortality	14 (5.6%)	14 (5.6%)	1.00 (0.44–2.24)	0.850
1-year mortality	16 (6.4%)	18 (7.2%)	0.89 (0.42–1.85)	0.864
**Secondary Outcomes**	**Non-IABP**	**IABP**	**OR (95% CI)**	***p***-**value**
Renal replacement therapy	17 (6.8%)	12 (4.8%)	0.71 (0.31–1.57)	0.458
Cerebrovascular accidents	10 (4.0%)	3 (1.2%)	0.30 (0.05–1.17)	0.096
Severe respiratory disorder^a^	11 (4.4%)	4 (1.6%)	0.36 (0.08–1.23)	0.121
Disabling complications^b^	29 (11.6%)	14 (5.6%)	0.48 (0.24–0.94)	0.033
Septicaemia	5 (2.0%)	8 (3.2%)	1.60 (0.46–6.22)	0.579
Infection^c^	26 (10.4%)	31 (12.4%)	1.20 (0.69–2.09)	0.600
**Secondary Outcome**	**Non-IABP**	**IABP**	**Mean Difference (95%CI)**	***p***-**value**
Post-operative length of stay (days) [IQR]	8.0 [6.0–12.0]	9.0 [7.0–14.8]	1.32 (-0.82–3.47)	0.225

^a^ Severe respiratory disorder secondary to pulmonary embolism, pulmonary edema, pneumonia, acute respiratory distress syndrome, or respiratory failure requiring ventilation

^b^ Defined as debilitating complications causing severe disability, consisting of cerebrovascular stroke, coma, renal insufficiency requiring renal replacement therapy, and severe respiratory disorders

^c^ Defined as an infection of the leg, mediastinum, sternum, urinary tract, or septicemia

#### Secondary outcomes

In Table 2, IABP-treated patients had a lower likelihood of disabling complications (OR = 0.48, 95%CI: 0.24–0.94) without observed IABP site-related infections. The IABP group showed fewer instances of renal insufficiency requiring RRT, cerebrovascular accidents, disabling complications, and severe respiratory disorders (Fig. 3A). In the multivariate analysis (Fig. 3B), IABP use was associated with decreased risk of severe respiratory disorders (OR: 0.10, 95%CI: 0.01–0.50, P-value = 0.011), lower 30-day and 90-day mortality, and improved outcomes for 1-year mortality, RRT, cerebrovascular accidents, severe respiratory disorders, and disabling complications.

## Discussion

A lower threshold for initiating IABP support was observed due to the higher risk profile of patients undergoing CABG, with 31% utilizing IABP. While IABP showed no survival benefit in high-risk (EuroSCORE II > 5.018%) patients, it did reduce the risk of disabling complications and short-term mortality, a new observation not highlighted in previous studies. Despite the absence of a long-term mortality reduction, the improvement in short-term outcomes justifies IABP usage, as it significantly enhances patients’ quality of life [4, 20].

IABP improves patient outcomes by reducing afterload via enhancing diastolic and lowering systolic aortic pressures. This results in lowered left ventricular wall stress and, hence, decreased demand for oxygen by the myocardium. Moreover, IABP concomitantly improves cardiac output by raising cardiac stroke volume, especially in patients with impaired left ventricular function. This, in turn, increases renal blood flow and improves renal function [21–24]. However, there is still much controversy surrounding the effects of pre-operative IABP implantation on post-operative outcomes. While many studies have pointed out the strong association between pre-operative IABP insertion and high postoperative mortality and morbidity [9, 11], the increased adverse events might be attributed to the presence of confounders.

The use of the IABP significantly improves the clinical outcomes of coronary patients, especially those with very low LVEF. One of the strongest indications for employing a preoperative IABP in patients undergoing CABG is a low ejection fraction, specifically an EF less than 30% [13, 17]. In our study, we observed that among both the non-IABP and IABP groups, both before and after PSM, the ejection fraction remained above 30%. This suggests that while preoperative IABP may be beneficial for patients with compromised cardiac function, even those with ejection fractions above 30% may still derive benefits from its use in the perioperative period.

With reference to the data displayed in Table 1, it becomes apparent that a considerable proportion of patients in the IABP group presented with peripheral vascular disease, extracardiac arteriopathy, or extensive atherosclerosis, mirroring findings observed in various other studies within the field. These findings are consistent with those reported in numerous other studies within the field. Patients with such comorbidities are known to be at elevated risk for peripheral limb complications, including limb ischemia, IABP site infection, thrombosis, and the requirement for further interventions, often resulting in significant disability [24, 25]. However, our study, after meticulous PSM, did not reveal a heightened incidence of such complications. This could be attributed to the robust methodology employed in our large-scale propensity matching process and the comprehensive analysis of an expanded patient cohort.

Conflicting results in studies may stem from significant heterogeneity between non-IABP and IABP groups, with surgeons favorably selecting higher pre-operative risk patients for IABP insertion, introducing selection bias [4]. Robust randomized control trials or propensity-score matching studies can mitigate this bias. Lack of consensus in the surgical community on the definition of high-risk patients further complicates the issue. While our study used EuroSCORE II > 5.018%, others defined high-risk patients using various clinical variables [16, 26].

Notably, the generalizability of IABP effects from studies like SHOCK II to bypass surgery patients is questioned [20]. Future research should focus on establishing a standardized criterion for defining high-risk patients benefiting from pre-operative IABP. Additionally, population and demographic factors, particularly in mixed Asian populations, remain understudied and may contribute to varied outcomes.

### Limitation

This study has limitations. Firstly, reliance on surgical registry data limits access to specific clinical details. Secondly, despite an 8-year duration and over 2900 patients, the small number of pre-operative IABP patients and low event rate may yield statistically insignificant secondary outcomes. Thirdly, Preoperative IABP patients were sicker, with more urgent CABG and higher LMS CAD rates. Post-matching, similar LMS CAD and urgent CABG rates seem improbable, likely explaining better IABP outcomes. Residual variables may confound results despite rigorous matching. Lastly, as a single-center study, variations in IABP practices across centers were not considered. A large-scale multi-center randomized control trial is needed for comprehensive validation.

## Conclusion

Utilizing pre-operative IABP in high-risk patients (EuroSCORE II > 5.018%) may lead to a reduction in 30-day and 90-day mortality rates, while also being associated with a decreased occurrence of severe respiratory complications. This underscores the potential benefits for high-risk patients in terms of improving short-term outcomes. However, it’s noteworthy that the use of IABP is significantly associated with disabling complications. The approach to preoperative IABP insertion in high-risk CABG patients may differ among centers, necessitating large-volume randomized controlled trials.

### Electronic supplementary material

Below is the link to the electronic supplementary material.

Supplementary Material 1

Supplementary Material 2

## Data Availability

The authors confirm that the data supporting the findings of this study are available within the article and its supplementary materials. Raw data were generated at Cardiac Surgery Research Laboratory at the National University of Singapore. Derived data supporting the findings of this study are available from the corresponding author on request.

## Declarations

### Clinical registration number

DSRB:2016/01070

## Abbreviations

BMI: Body mass index
CABG: Coronary artery bypass grafting
CHAID: Chi-square automatic interaction detection
DSRB: Domain specific review board
EuroSCORE: European system for cardiac operative risk evaluation
IABP: Intra-aortic balloon pump
NUH: National University Hospital
OR: Odds ratio
PCI: Percutaneous intervention
PSM: Propensity score matching
RRT: Renal replacement therapy
SHOCK: Should we emergently revascularize occluded coronaries for cardiogenic shock
