# Supplementary figures and images for: Is preoperative IABP insertion significantly reducing postoperative complication in augmented high-risk coronary artery bypass grafting patients?
Source: J Cardiothorac Surg. 2024 Jun 24;19:363. doi: 10.1186/s13019-024-02925-2 (PMC11194871; doi:10.1186/s13019-024-02925-2)

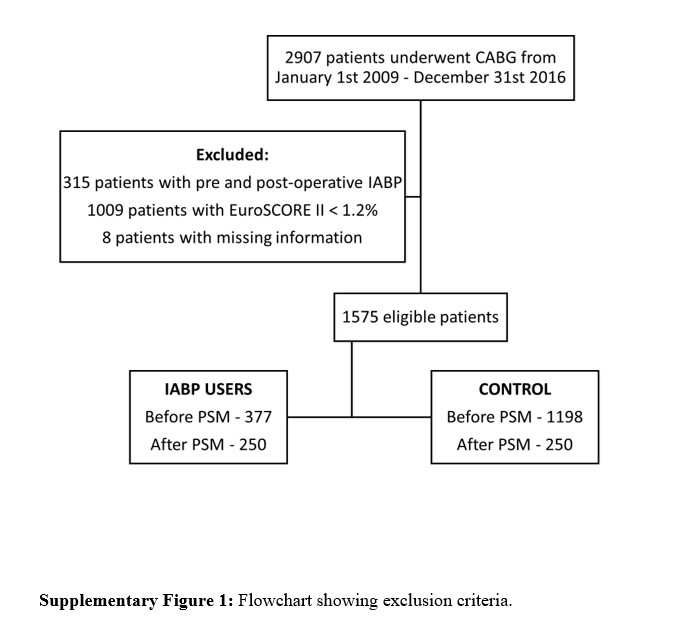

Supplement: Supplementary file 1 — Supplementary Material 1 [file 13019_2024_2925_MOESM1_ESM.jpg]

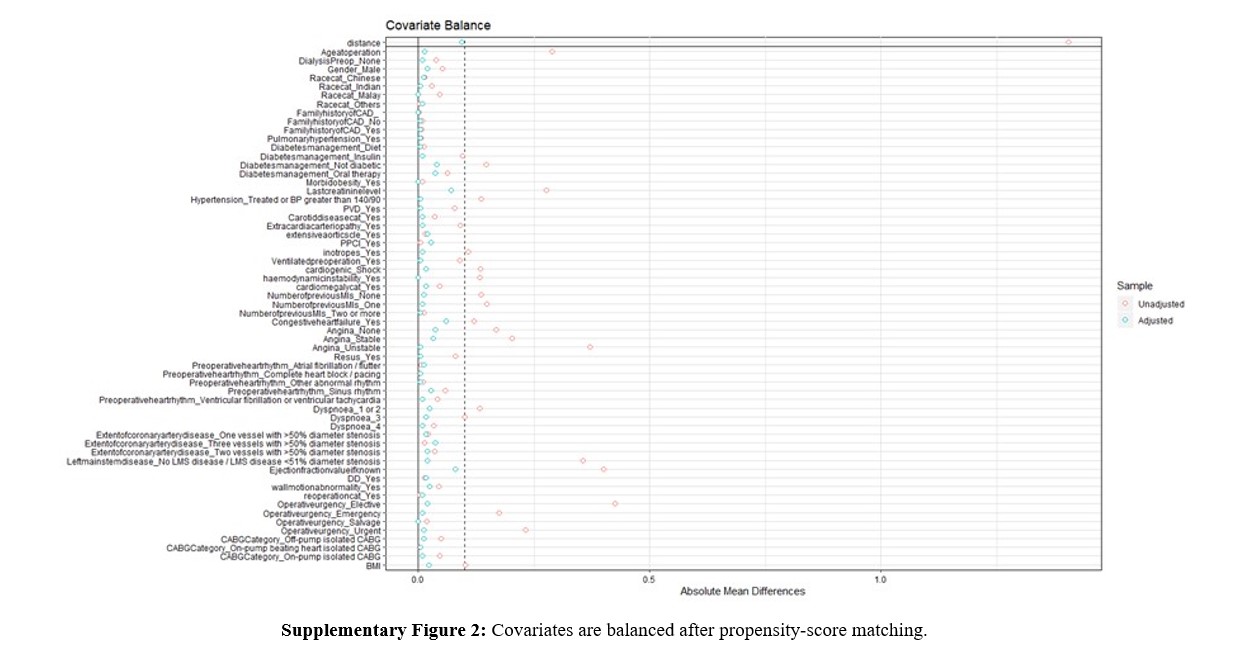

Supplement: Supplementary file 2 — Supplementary Material 2 [file 13019_2024_2925_MOESM2_ESM.jpg]
